# Integrating a Host-Protein Bacterial/Viral Test into the Emergency Department Sepsis Alert Order Set Is Associated with Changes in Sepsis Category Distribution and Patient Management: A Pre/Postimplementation Study

**DOI:** 10.1097/CCE.0000000000001523

**Published:** 2026-11-13

**Authors:** Marci O’Driscoll, Suzanne Silbert, Tashkin Tokaria, Shannon Young, Ashkon Chamani, Daniel Wind, Kimberly Munoz, Jason W. Wilson

**Affiliations:** 1 Esoteric Testing Laboratory, Tampa General Hospital, Tampa, FL.; 2 Department of Emergency Medicine, Tampa General Hospital, Tampa, FL.; 3 Department of Emergency Medicine, Morsani College of Medicine, University of South Florida. Tampa, FL.

**Keywords:** bacterial vs. viral, emergency medicine, host response, MeMed BV, sepsis alerts

## Abstract

**OBJECTIVES:**

To evaluate the impact of implementing a host-protein bacterial/viral test in the emergency department (ED) sepsis alert order set.

**DESIGN:**

Pre/postimplementation study. Host-protein testing was automatically ordered both pre- and postimplementation as part of the ED sepsis alert order set; test results were accessible to treating clinicians only postimplementation. The pre- (September 2024 to December 2024) to postimplementation (January 2025 to March 2025) transition also involved education on the host-protein test to ED clinicians. The primary outcome was patient classification into Surviving Sepsis Campaign (SSC) sepsis categories (definite/probable vs. possible vs. unlikely sepsis).

**SETTING:**

ED of an academic medical center.

**PATIENTS:**

Adults who triggered the ED sepsis alert order set.

**INTERVENTIONS:**

Host-protein bacterial/viral test (MeMed BV) automatically ordered in ED sepsis alert order set. Test result was only visible to clinicians postimplementation.

**MEASUREMENTS AND MAIN RESULTS:**

There were 893 eligible patients; 505 patients in the preimplementation cohort and 388 in the postimplementation cohort. The distribution of SSC sepsis categories differed between pre- and postimplementation cohorts (χ^2^(2) = 8.73; *p* = 0.013). The difference was primarily driven by a larger proportion of patients classified as unlikely sepsis (from 32.5% to 41.1%; *p* = 0.009) and a smaller proportion classified as possible sepsis (from 18.4% to 13.2%; *p* = 0.043). Among unlikely sepsis patients, host-protein test implementation was associated with ordering fewer cefepime doses (from 3.3 to 2.2; *p* = 0.044) and a lower hospitalization rate (from 92.1% to 79.9%; *p* = 0.002).

**CONCLUSIONS:**

These findings support an association between host-protein testing for patients triggering ED sepsis alerts and the distribution of possible vs. unlikely sepsis cases. Multicenter clinical utility studies are warranted.

**KEY POINTS**

**Question**: Does integrating host-protein bacterial/viral testing into an emergency department (ED) sepsis order set impact sepsis category classification and patient management?

**Findings:** Test implementation was associated with an increased proportion of unlikely sepsis cases (32.5 to 41.1%) and reduced proportion of possible cases (18.4 to 13.2%); the proportion of definitive/probable sepsis cases was unchanged. Unlikely sepsis patients managed postimplementation were ordered fewer doses of cefepime, and a reduced proportion were hospitalized.

**Meaning**: These findings provide preliminary evidence that integrating a host-protein bacterial/viral test into an ED sepsis alert order set may support sepsis classification and patient management. Prospective multicenter evaluation is warranted.

Timely antibiotic treatment can prevent deterioration of bacterial infection into sepsis and septic shock (1). Time sensitivity, along with diagnostic uncertainty, drives antibiotic ordering for patients triggering sepsis alerts at the emergency department (ED), leading to treatment of patients who do not have a bacterial infection (2, 3). The 2026 Surviving Sepsis Campaign (SSC) guidelines introduced a diagnostic framework classifying patients as having definitive/probable, possible, or unlikely sepsis to guide the decision window for antibiotic ordering (4). The SSC panel found insufficient real-world evidence to recommend host response testing for ruling out bacterial infection (4, 5).

MeMed BV (MMBV) is a Food and Drug Administration-cleared host-protein test integrating three circulating immune proteins (Tumor necrosis factor-related apoptosis-inducing ligand [TRAIL], Interferon gamma-induced protein 10 [IP-10], and C-reactive protein [CRP]) to generate a bacterial likelihood score from 0 to 100 within 15 minutes. Across studies in adults and children, the host-protein test demonstrated sensitivity and specificity exceeding 90% and a negative predictive value of 95–98% for distinguishing bacterial infection, outperforming individual biomarkers including procalcitonin (6, 7). Result availability led to reduced antibiotic orders with a decrease in return hospitalizations for respiratory infection in a previous study (8). The test has not yet been evaluated in real-world practice within an ED sepsis order set.

Here we report a pre-/postimplementation study at an academic hospital designed to evaluate the impact of host-protein test integration into the ED sepsis alert order set. Among adult patients triggering a sepsis alert, we assessed whether implementation was associated with altered patient distribution across sepsis classification categories and examined antibiotic ordering and hospital admission rates per category. This study was intended to generate preliminary evidence to inform the design of future prospective multicenter comparator studies.

## MATERIALS AND METHODS

### Ethics

Institutional Review Board (IRB) approval was obtained at Tampa General Hospital (TGH; IRB number: STUDY006477; Title: MeMed Review; February 6, 2024), with a waiver of informed consent granted. The study was conducted in accordance with the Declaration of Helsinki.

### Study Design and Setting

TGH is an academic medical center with 85,000 annual visits. This was a single-center pre/postimplementation study evaluating the clinical impact of host-protein test integration into the ED electronic sepsis alert order set. A structured retrospective chart review was performed by trained abstractors.

Host-protein testing was automatically ordered pre- and postimplementation; test results were accessible to treating clinicians in the electronic health record only postimplementation. The preimplementation (September 2024 to December 2024; 4 mo) to postimplementation (January 2025 to March 2025; 3 mo) transition included education specific to the host-protein test (score interpretation, intended use, and order set integration) delivered via ED weekly emails and monthly ED meetings. No general education on sepsis recognition or management was provided.

### Patient Selection

Adult patients who triggered an ED sepsis alert with a host-protein result were eligible. The ED sepsis alert is triggered automatically by two or more Severe Inflammatory Response Syndrome (SIRS) criteria or manually based on clinical suspicion; these could not be distinguished in our data. Patients were excluded if they had host-protein results without a corresponding sepsis alert or were missing a valid host-protein result. One patient missing a discharge diagnosis was excluded. Since age data were banded in decades (i.e., 0–10 yr, 10–20 yr, etc.), to ensure inclusion of adults, the eligibility criterion was age older than 20 years.

### Implemented Host-Protein Test

MMBV (MeMed, Andover, MS) was run on the MeMed Key platform in the Esoteric laboratory with standard opening hours from 7:00 to 22:00. Validated score thresholds classify results as viral/nonbacterial (< 35), equivocal (35–65), or bacterial including coinfection (> 65) (6, 7).

### Outcomes and Statistical Analysis

Each patient’s free-text clinical discharge diagnosis was assigned by trained abstractors to a standardized categorical diagnosis field, which was then mapped to SSC 2026 sepsis categories. “Sepsis of any infectious etiology” (including bacterial, viral, unspecified, and mixed) was mapped to “definitive/probable sepsis,” “bacterial infection not meeting sepsis criteria” to “possible sepsis,” and “viral infection or noninfectious illness meeting SIRS criteria” to “unlikely sepsis.” The primary outcome was patient sepsis classification into SSC 2026 categories (definitive/probable, possible, and unlikely) compared across pre- and postimplementation cohorts. Secondary outcomes included antibiotic ordering rate, hospital admission rate, mean doses of cefepime and vancomycin ordered, in-hospital mortality, and length of stay, assessed within each sepsis category. Analyses were conducted across the full cohort and stratified by immune status (immunocompetent vs. immunosuppressed). Immunosuppressed was defined as active malignancy, solid organ or hematopoietic stem cell transplant, and/or receipt of immunosuppressive therapy.

Continuous variables were reported as medians with interquartile ranges and compared using the Mann-Whitney *U* test; categorical variables were reported as proportions compared using chi-square and Fisher exact test. A *p* value of less than 0.05 was considered statistically significant. Statistical analyses were performed using Python version 3.11.12 (Python Software Foundation, Beaverton, OR).

## RESULTS

Of 916 eligible patients, 893 were included; the preimplementation cohort comprised 505 patients, and the postimplementation cohort comprised 388 patients (**Fig. 1**). Age, sex, and WBC counts were comparable between cohorts (**Table 1**).

**TABLE 1.** Patient Characteristics and Outcomes Per Sepsis Category for the Study Cohort

Characteristics	Definitive/Probable Sepsis		Possible Sepsis		Unlikely Sepsis	
	Preimplementation Cohort	Postimplementation Cohort	Preimplementation Cohort	Postimplementation Cohort	Preimplementation Cohort	Postimplementation Cohort
All patients (*n* = 892)	*n* = 248	*n* = 177	*n* = 93	*n* = 51	*n* = 164	*n* = 159
Demographics						
Sex at birth, male, *n* (%)	134 (54.0)	87 (49.4)	51 (54.8)	23 (45.1)	87 (53.0)	78 (49.1)
Age group, *n* (%)						
Age, ≤ 50	75 (30.2)	62 (35.2)	40 (43.0)	19 (37.3)	55 (33.5)	54 (34.0)
Age, 51–60	49 (19.8)	29 (16.5)	16 (17.2)	6 (11.8)	27 (16.5)	33 (20.8)
Age, 61–70	47 (19.0)	39 (22.2)	14 (15.1)	11 (21.6)	45 (27.4)	34 (21.4)
Age, 71–80	53 (21.4)	31 (17.6)	18 (19.4)	7 (13.7)	23 (14.0)	24 (15.1)
Age, > 80	24 (9.7)	15 (8.5)	5 (5.4)	8 (15.7)	14 (8.5)	14 (8.8)
Immune status, *n* (%)						
Uncertain	12 (4.8)	6 (3.4)	5 (5.4)	1 (2.0)	7 (4.3)	6 (3.8)
Immunocompetent	118 (47.6)	111 (62.7)	57 (61.3)	30 (58.8)	79 (48.2)	95 (59.7)
Immunosuppressed	118 (47.6)	60 (33.9)	31 (33.3)	20 (39.2)	78 (47.6)	58 (36.5)
Diagnostics performed						
WBC, cells/µL, median (IQR)	12.8 (8.0–16.8)	13.6 (9.4–18.6)	11.1 (7.3–15.0)	12.3 (8.7–14.2)	10.9 (7.1–15.1)	10.5 (7.9–14.5)
Chest radiograph ordered, *n* (%)	218 (87.9)	150 (84.7)	67 (72.0)	35 (68.6)	133 (81.1)	139 (87.4)
Additional bacterial testing^a^, *n* (%)	214 (86.3)	152 (85.9)	75 (80.6)	38 (74.5)	97 (59.1)	89 (56.0)
Patient outcomes						
Admission rate, *n* (%)	243 (98.0)	170 (96.0)	83 (89.2)	42 (82.4)	151 (92.1)	127 (79.9)
Hospital length of stay, d, median (IQR)	7.0 (4.0–12.0)	7.0 (4.0–11.0)	5.0 (3.0–10.0)	6.0 (3.0–8.0)	5.0 (3.0–9.0)	5.0 (3.0–8.0)
Antibiotics ordered, *n* (%)	248 (100.0)	177 (100.0)	92 (98.9)	47 (92.2)	161 (98.2)	138 (86.8)
Doses of vancomycin, mean (sd)	3.5 (8.2)	3.1 (6.2)	2.6 (5.0)	2.9 (8.2)	1.1 (2.5)	0.7 (2.3)
Doses of cefepime, mean (sd)	4.3 (6.3)	3.8 (5.0)	4.0 (6.4)	3.2 (4.6)	3.3 (5.4)	2.2 (3.9)
In-hospital mortality, *n* (%)	14 (5.7)	16 (9.0)	2 (2.2)	1 (2.0)	1 (0.6)	4 (2.5)
Follow-up outcome for nonadmitted patients	*n* = 5	*n* = 7	*n* = 10	*n* = 9	*n* = 13	*n* = 32
Follow-up visit within next 7 d, *n* (%)	0 (0.0)	1 (14.3)	2 (20.0)	3 (33.3)	2 (15.4)	5 (15.6)
Follow-up visit with hospitalization, *n* (%)	0 (0.0)	1 (14.3)	2 (20.0)	2 (22.2)	1 (7.7)	0 (0.0)
Antibiotic prescribed at follow-up visit, *n* (%)	0 (0.0)	0 (0.0)	2 (20.0)	3 (33.3)	2 (15.4)	0 (0.0)
30-d mortality, *n* (%)	0 (0.0)	0 (0.0)	0 (0.0)	0 (0.0)	0 (0.0)	0 (0.0)

IQR = interquartile range.

a Additional bacterial testing comprised multiple microbiological sampling (e.g., blood, urine, bronchoalveolar lavage, …) and testing (e.g., culture, polymerase chain reaction, …) methodologies aimed at identifying methicillin-resistant *Staphylococcus aureus*/methicillin-sensitive *Staphyolococcus aureus*, sexually transmitted infections, urinary tract infections, gastrointestinal infections, atypical bacterial infections, *Clostridioides difficile*, and other bacterial-induced infections.

**Figure 1.**
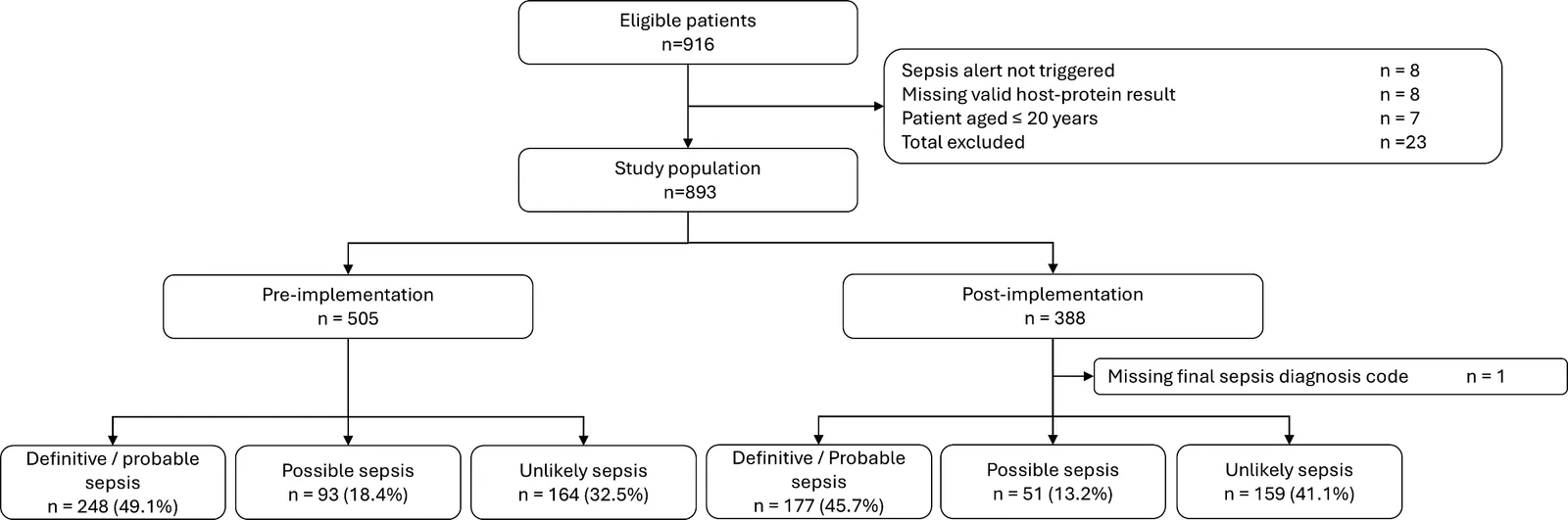
Study flow diagram showing the preimplementation and postimplementation cohorts and the distribution of SSC categories per cohort.

Host-protein test implementation was associated with a significant shift in the distribution of patients across sepsis categories (χ^2^(2) = 8.73; *p* = 0.0127; Fig. 1 and Table 1). The difference was primarily driven by a larger proportion of patients classified as unlikely sepsis (from 32.5% to 41.1%; *p* = 0.009) and a smaller proportion classified as possible sepsis (from 18.4% to 13.2%; *p* = 0.043). The proportion classified as definitive/probable sepsis did not change (49.1% vs. 45.7%; *p* = 0.344). Overall, the rate of any antibiotic ordered was lower postimplementation among patients with viral/nonbacterial scores (from 98.9% to 82.4%; *p* < 0.001), with no observed effect among patients with equivocal/bacterial scores.

Within the unlikely sepsis category, host-protein test implementation was associated with significantly fewer mean cefepime doses ordered (from 3.3 to 2.2; relative reduction 33.3%; *p* = 0.044), and a lower hospital admission rate (from 92.1% to 79.9%; relative reduction 13.2%; *p* = 0.002). Among those with an unlikely sepsis classification who were discharged with fewer antibiotics ordered, the rate of return visits within 7 days was unchanged following implementation (15.4 to 15.6%), with no postdischarge deaths within 30 days in either period (Table 1). In-hospital mortality was 0.6% and 2.5% in the pre- and postimplementation cohorts, respectively (*p* = 0.166). In patients with definitive/probable sepsis, antibiotic ordering was 100% pre- and postimplementation and there were no differences in other outcomes.

There was a greater proportion of immunocompetent patients postimplementation (60.8% vs. 50.3%; *p* = 0.002). Subgroup analysis demonstrated that the distributional shift in sepsis categories was observed in the immunocompetent subgroup (*n* = 490; **Supplemental Table 1**, https://links.lww.com/CCX/B707; χ^2^(2) = 9.42; *p* = 0.009) and was driven by a smaller proportion of possible sepsis cases (from 22.4% to 12.7%; relative reduction 43.3%; *p* = 0.006) and a larger proportion of unlikely sepsis cases (from 31.1% to 40.3%; relative increase 29.6%; *p* = 0.035). Implementation was not associated with a difference in distribution across sepsis categories in the immunosuppressed subgroup (*n* = 365; *p* > 0.05). Host-protein test implementation was associated with reduced antibiotic exposure and reduced hospitalization among unlikely sepsis patients only in the immunocompetent subgroup.

## DISCUSSION

Host-protein test integration into an ED sepsis order set was associated with a significant shift from possible toward unlikely sepsis classification, with reductions in cefepime exposure and hospital admission in the unlikely sepsis group. Critically, antibiotic ordering in definitive/probable sepsis remained 100% pre- and postimplementation, suggesting that observed management changes were not applied to high-acuity patients. To our knowledge, these findings represent the first implementation data suggesting a host response bacterial/viral test may influence sepsis classification.

Our findings are consistent with the interpretation that host-protein bacterial/viral testing helps borderline possible sepsis cases to be categorized as unlikely sepsis and managed accordingly (9). The observed shift in sepsis category distribution was present within the immunocompetent subgroup, indicating the findings are not solely attributable to immunosuppression differences between cohorts. Importantly, the association between host-protein testing and antibiotic stewardship plus reduced admissions accords with observations in a randomized trial in suspected lower respiratory infection (8) and a real-world ED blood-culture cohort (10).

A study strength is the straightforward workflow, with the test auto-ordered in the ED sepsis alert order set. Another strength is that the primary outcome is based on the treating team’s clinical diagnosis recorded in the electronic health record, rather than a determination made by the investigators or abstractors. This diagnosis may be informed by the host-protein test result. Importantly, the accompanying reduction in antibiotic ordering specific to viral/nonbacterial host-protein test scores supports a change in clinical management rather than documentation alone.

The study has limitations. The findings are from a single center and warrant replication at additional sites to ensure generalizability. The SIRS-based alert may capture a broader population than settings with more restrictive criteria. As for all pre/post studies, it is susceptible to temporal confounding, with the implementation periods spanning different phases of the respiratory virus season. Importantly, the antibiotic ordering reduction specifically among viral/nonbacterial scores is unlikely to reflect a seasonal effect, which, if anything, would be expected to increase prescribing given winter respiratory pressure and coinfection uncertainty. A Hawthorne effect is also an unlikely explanation: enhanced concern for sepsis would increase, not decrease, antibiotic ordering. Lastly, the low number of return visits and mortality events limits the interpretation of these outcomes; notably, no deaths were reported among discharged patients. In conclusion, implementation of auto-ordering of a rapid host-protein test was associated with changes in sepsis classification and patient management. Multicenter studies are warranted to further establish host response testing in safe management of sepsis patients.

## ACKNOWLEDGMENTS

We thank our colleagues for data analysis support: Tanya M. Gottlieb, Boris Lebedenko, Jeroen Stas, and Roy Navon.

## Supplementary Material



## References

[1] Meyer NJ Prescott HC: Sepsis and septic shock. N Engl J Med 2024; 391:2133–2146 10.1056/NEJMra2403213 39774315

[2] Shappell CN Yu T Klompas M: Frequency of antibiotic overtreatment and associated harms in patients presenting with suspected sepsis to the emergency department: A retrospective cohort study. Clin Infect Dis 2025; 80:1197–1207 10.1093/cid/ciaf118 40231968

[3] Singer M Angus DC Annane D: Sepsis. Lancet 2026; 407:1276–1288 10.1016/S0140-6736(25)02422-5 41765030

[4] Prescott HC Antonelli M Alhazzani W: Surviving sepsis campaign: International guidelines for management of sepsis and septic shock 2026. Crit Care Med 2026; 54:725–812 10.1097/CCM.0000000000007075 41869847

[5] Deshpande B Horowitz JK McLaughlin E: C36-12 uptake of rapid diagnostic aids for sepsis across Michigan hospitals. Am J Respir Crit Care Med 2026; 212(Suppl 1):aamag162.6209

[6] Bachur RG Kaplan SL Arias CA: A rapid host-protein test for differentiating bacterial from viral infection: Apollo diagnostic accuracy study. J Am Coll Emerg Physicians Open 2024; 5:e13167 10.1002/emp2.13167 PMC11077430 38721037

[7] Allen C Deanehan JK Dotan Y: Development of a reference standard to assign bacterial versus viral infection etiology using an all-inclusive methodology for comparison of novel diagnostic tool performance. Clin Infect Dis 2025; 80:735–743 10.1093/cid/ciae656 PMC12043069 39750735

[8] Singer AJ Hollander JE Kean ER: Effect of host-protein test (TRAIL/IP-10/CRP) on antibiotic prescription and emergency department or urgent care center return visits: The JUNO pilot randomized controlled trial. Acad Emerg Med 2025; 32:975–984 10.1111/acem.70031 PMC12435126 40251855

[9] Kalmovich B Rahamim-Cohen D Yehoshua I: Implementation of a rapid host-protein diagnostic test for distinguishing bacterial and viral infections in adults presenting to urgent care centers: A pragmatic cohort study. BMC Med 2025; 23:63 10.1186/s12916-025-03903-8 PMC11792296 39901146

[10] LoVerde A Ghitan M Bodenstein H: Real-world study assessing sensitivity and clinical utility of a host-protein test in adult emergency department patients with blood culture ordered. J Am Coll Emerg Physicians Open 2025; 6:100255 10.1016/j.acepjo.2025.100255 PMC12514503 41081286

