# Supplementary material for: Integrating a Host-Protein Bacterial/Viral Test into the Emergency Department Sepsis Alert Order Set Is Associated with Changes in Sepsis Category Distribution and Patient Management: A Pre/Postimplementation Study
Source: Crit Care Explor. 2026 Nov 13;8(11):e1523. doi: 10.1097/CCE.0000000000001523 (PMC13766560; doi:10.1097/CCE.0000000000001523)
Supplement: Supplementary file 1 [file cc9-8-e1523-s001.pdf]

Supplemental Table 1. Patient characteristics and outcomes per sepsis category for the immunocompetent subgroup

Characteristics	Definitive / Probable sepsis		Possible sepsis		Unlikely sepsis	
	Pre-implementation cohort	Post-implementation cohort	Pre-implementation cohort	Post-implementation cohort	Pre-implementation cohort	Post-implementation cohort
Immunocompetent patients (n = 490)	n = 118	n = 111	n = 57	n = 30	n = 79	n = 95
**Demographics**						
Sex at birth, male: n (%)	63 (53.4%)	49 (44.5%)	29 (50.9%)	15 (50.0%)	41 (51.9%)	43 (45.3%)
Age group; n (%)						
Age ≤ 50	37 (31.4%)	44 (40.0%)	29 (50.9%)	12 (40.0%)	30 (38.0%)	38 (40.0%)
Age: 51-60	23 (19.5%)	13 (11.8%)	10 (17.5%)	3 (10.0%)	8 (10.1%)	18 (18.9%)
Age: 61-70	20 (16.9%)	22 (20.0%)	8 (14.0%)	5 (16.7%)	22 (27.8%)	17 (17.9%)
Age: 71-80	24 (20.3%)	20 (18.2%)	5 (8.8%)	5 (16.7%)	10 (12.7%)	12 (12.6%)
Age: > 80	14 (11.9%)	11 (10.0%)	5 (8.8%)	5 (16.7%)	9 (11.4%)	10 (10.5%)
Immune status; n (%)						
Uncertain	0 (0%)	0 (0%)	0 (0%)	0 (0%)	0 (0%)	0 (0%)
Immunocompetent	118 (100.0%)	111 (100.0%)	57 (100.0%)	30 (100.0%)	79 (100.0%)	95 (100.0%)
Immunosuppressed	0 (0%)	0 (0%)	0 (0%)	0 (0%)	0 (0%)	0 (0%)
**Diagnostics performed**						
WBC, cells/µl; median (IQR)	13.3 (9.7, 17.2)	13.9 (10.6, 18.0)	12.4 (8.2, 16.1)	12.3 (8.5, 14.5)	13.4 (9.0, 16.0)	11.3 (8.6, 14.7)
Chest X-ray ordered; n (%)	103 (87.3%)	91 (82.0%)	37 (64.9%)	20 (66.7%)	61 (77.2%)	81 (85.3%)
Additional bacterial testing*; n (%)	100 (84.7%)	94 (84.7%)	48 (84.2%)	21 (70.0%)	42 (53.2%)	49 (51.6%)
**Patient outcomes**						
Admission rate; n (%)	114 (96.6%)	107 (96.4%)	49 (86.0%)	23 (76.7%)	70 (88.6%)	72 (75.8%)
Hospital length of stay, days; median (IQR)	6.5 (4.0, 12.0)	6.0 (4.0, 11.0)	5.0 (3.0, 12.0)	5.0 (3.0, 9.0)	5.0 (3.0, 8.8)	4.0 (3.0, 7.2)
Antibiotics ordered; n (%)	118 (100.0%)	111 (100.0%)	56 (98.2%)	27 (90.0%)	79 (100.0%)	80 (84.2%)
Doses of vancomycin; mean (SD)	4.3 (10.7)	3.2 (6.5)	1.7 (3.4)	4.2 (10.4)	1.4 (3.2)	0.6 (1.9)
Doses of cefepime; mean (SD)	3.8 (6.3)	3.7 (5.1)	3.9 (6.7)	2.6 (4.2)	3.4 (5.6)	1.6 (3.2)
In hospital mortality; n (%)	6 (5.1%)	8 (7.2%)	0 (0.0%)	0 (0.0%)	1 (1.3%)	2 (2.1%)
**Follow-up outcome non-admitted patients**	n=4	n=4	n=8	n=7	n=9	n=23
Follow-up visit within next 7 days; n (%)	0 (0.0%)	0 (0.0%)	1 (12.5%)	2 (28.6%)	2 (22.2%)	5 (21.7%)
Follow-up visit with hospitalization; n (%)	0 (0.0%)	0 (0.0%)	1 (12.5%)	1 (14.3%)	1 (11.1%)	0 (0.0%)
Antibiotic prescribed at follow-up visit; n (%)	0 (0.0%)	0 (0.0%)	1 (12.5%)	2 (28.6%)	2 (22.2%)	0 (0.0%)

30-day mortality; n (%)	0 (0.0%)	0 (0.0%)	0 (0.0%)	0 (0.0%)	0 (0.0%)	0 (0.0%)

*Additional bacterial testing comprised multiple microbiological sampling (e.g., blood, urine, BAL, …) and testing (e.g., culture, PCR, …) methodologies aimed at identifying MRSA/MSSA; STIs; UTIs; GIs; Atypical bacterial infections; *C. difficile* ; and other bacterial induced infections.

With BAL, Bronchoalveolar Lavage; GIs; Gastrointestinal infections; IQR, Interquartile Range; MRSA, methicillin-resistant *S. aureus*; MSSA, methicillin-sensitive *S. aureus*; SD, Standard deviation; STIs, sexual-transmitted infections; UTIs, Urinary tract infections; and WBC, White Blood cells.
